# Inferring Potential microRNA-microRNA Associations Based on Targeting Propensity and Connectivity in the Context of Protein Interaction Network

**DOI:** 10.1371/journal.pone.0069719

**Published:** 2013-07-16

**Authors:** Jie Sun, Meng Zhou, Haixiu Yang, Jiaen Deng, Letian Wang, Qianghu Wang

**Affiliations:** College of Bioinformatics Science and Technology, Harbin Medical University, Harbin, China; Cankiri Karatekin University, Turkey

## Abstract

MicroRNAs (miRNAs) are a group of small non-coding RNAs that play important regulatory roles at the post-transcriptional level. Although several computational methods have been developed to compare miRNAs, it is still a challenging and a badly needed task with the availability of various biological data resources. In this study, we proposed a novel graph theoretic property based computational framework and method, called miRFunSim, for quantifying the associations between miRNAs based on miRNAs targeting propensity and proteins connectivity in the integrated protein-protein interaction network. To evaluate the performance of our method, we applied the miRFunSim method to compute functional similarity scores of miRNA pairs between 100 miRNAs whose target genes have been experimentally supported and found that the functional similarity scores of miRNAs in the same family or in the same cluster are significantly higher compared with other miRNAs which are consistent with prior knowledge. Further validation analysis on experimentally verified miRNA-disease associations suggested that miRFunSim can effectively recover the known miRNA pairs associated with the same disease and achieve a higher AUC of 83.1%. In comparison with similar methods, our miRFunSim method can achieve more effective and more reliable performance for measuring the associations of miRNAs. We also conducted the case study examining liver cancer based on our method, and succeeded in uncovering the candidate liver cancer related miRNAs such as miR-34 which also has been proven in the latest study.

## Introduction

MicroRNAs (miRNAs), ∼ 22 nucleotides (nt) in length, are a major class of short endogenous non-coding RNA (ncRNA) molecules that play important regulatory roles at the post-transcriptional level by targeting mRNAs for cleavage or translational repression [1], [2]. Since the discovery of miRNA molecules *lin-4* and *let-7* in 1993 in *Caenorhabditis elegans* through forward genetic screens [3], more and more novel miRNAs have been identified in almost all metazoan genomes, including worms, flies, plants and mammals by forward genetics, direct cloning, high-throughput sequencing technology and bioinformatics approaches [4], [5], [6]. To date, 1600 miRNAs of the human genome have been annotated in the latest version of the miRBase [7].

During the past several years, many methods have been proposed to compare the functional similarities between different protein-coding genes for further better understanding of the underlying biological phenomena or discovering previously unknown gene functions [8], [9], [10], [11], [12]. With the growth of information on miRNAs, miRNAs have been shown as a group of important regulators to regulate basic cellular functions including proliferation, differentiation and death [13], [14], [15], [16]. However, the functions of most miRNAs remain unknown. Therefore, to better understand miRNAs and their roles in the underlying biological phenomena, biologists are paying more attention to compare miRNA genes and want to know the associations between them. For example, comparing similarities between miRNA with known molecular functions or associated with specific disease and that with unknown functions would allow us to infer potential functions for novel miRNAs, or help us to identify potential candidate disease-related miRNAs for guiding further biological experiments. However, until now, only several computational methods have been developed to meet the requirement [17], [18]. Therefore, comparing miRNAs is still a challenging and a badly needed task with the availability of various biological data resources.

Many studies have shown that the functions of miRNAs can be predicted or inferred by analyzing the properties of miRNA targets [19], [20], [21]. It has been reported that the targeting propensity of miRNA can be largely explained by the functional behavior of protein connectivity in the protein-protein interaction network (PPIN) [22], [23]. With the rapid advances in biotechnology, large-scale PPIN is currently available and is already rich enough to evaluate the relationship between miRNAs based on their targeting propensity in PPIN. Here, based on the above notion, we proposed a novel computational method, called miRFunSim, to quantify the associations between miRNAs in the context of protein interaction network. We evaluated and validated the performance of our miRFunSim method on miRNA family, miRNA cluster data and experimentally verified miRNA-disease associations. Further comparison analysis showed that our method is more effective and reliable as compared to other existing similar methods, and offers a significant advance in measuring the associations between miRNAs.

## Materials and Methods

### Construction of Integrated Human Protein Interaction Network

The high throughput protein-protein interaction data were obtained from Wang’s study [24] consisting of 69,331 interactions between 11,305 proteins, which integrated BioGRID [25], IntAct [26], MINT [27], HPRD [28] and by the Co-citation of text mining [29] databases and made further filtering to improve coverage and quality of PPIN and reduce false-positives produced by different prediction algorithms in different databases.

### Human miRNA Datasets

All known human miRNAs were from miRBase Sequence Database, release 16 (http://www.mirbase.org/) [30]. We used experimentally verified miRNA targets from TarBase which houses a manually curated collection of experimentally supported miRNA targets in several animal species [31] (File S1). The predicted miRNA targets were downloaded from starBase database which provides a comprehensive integrated miRNA-target map [32]. Because of the high false positive rate of predicted miRNA targets, we only chose miRNA targets predicted by at least three prediction algorithms with readNum > = 1 and biological complexity > = 1 (File S2). The genome coordinates of miRNAs were downloaded from miRBase Sequence Database, release 16 (http://www.mirbase.org/) [30]. Those miRNAs with pair-wise distance less than 10 kb were considered as clustered miRNAs. The high-quality experimentally verified miRNA-disease association data was retrieved from Jiang’s study [33].

### Statistical Analysis

The functional similarity score between miRNAs may be generated by chance. In order to take this effect into account and obtain the statistical significance of scores, we performed randomization test and repeated 1000 times. For each score, 1000 simulated miRNA pairs were generated and target genes of simulated miRNA pairs were randomly sampled from all human protein-coding genes keeping the same size as given miRNA pairs. Then the functional similarity scores between simulated miRNA pairs were recomputed for each simulated miRNA pair denoted SFSSM. M denoted the number of simulated miRNA pairs having an equal or larger SFSSM value than the true score. The estimate of the empirical statistical significance value, P-value, of true score was obtained as P = M/1001. The empirical P-value based on such randomizations represented the probability of obtaining a score greater than a given score by chance.

## Results and Discussion

### Overview of miRFunSim

**Figure 1:**
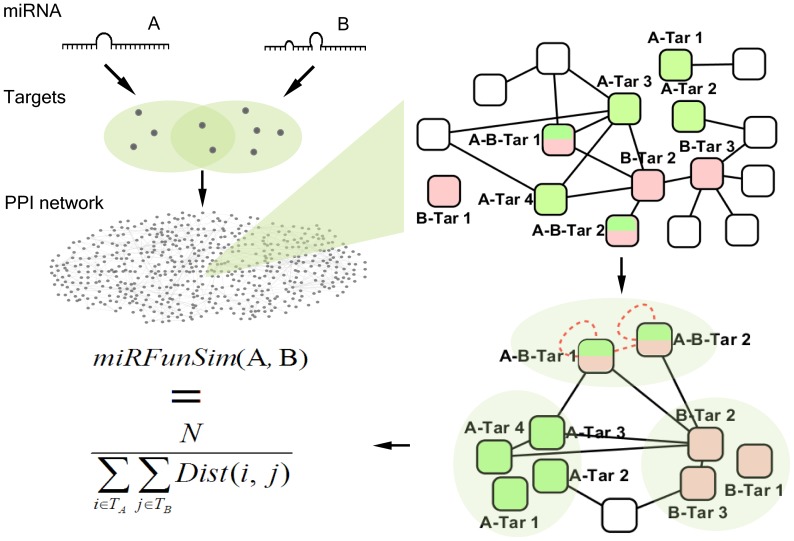
The schematic representation and overview of the miRFunSim method.



In this study, we developed a graph theoretic property based method, miRFunSim, to quantify the associations between two miRNA in the context of targets propensity in the protein-protein interaction network. A schematic representation of the miRFunSim method is shown in Figure 1. Initially, given two interested miRNAs, miRNA A and miRNA B, we evaluate the functional relationship between them using the protein interaction network. First, we obtain the target gene lists for each miRNA, which are denoted by *T_A_* and *T_B_* respectively. There may be existing common targets between *T_A_* and *T_B_*. Second, we map the target genes from these lists onto the integrated protein interaction network. Then, the protein interaction sub-network of target genes is generated from the integrated protein interaction network. Here, the targets of miRNA A and miRNA B are marked in red and green respectively in the protein interaction sub-network of target genes. The nodes colored by both of the red and green in the protein interaction sub-network are the common targets. Thirdly, the distance between two nodes which are from *T_A_* and *T_B_* respectively is the length of the shortest path and can be calculated based on the sub-network which reflects the protein connectivity and functional associations of targets. For the overlap of targets between *T_A_* and *T_B_*, we made two hypotheses: there exists a ring on the node that represents the common target, and there exists a shortcut between two targets except they are isolated. Based on the above hypotheses, we add the hypothetical edges into the protein interaction sub-network, where the new edges are marked in red. Finally, the functional similarity score between two miRNAs is defined as the reciprocal of average pair-wise distance between *T_A_* and *T_B_*, and is computed as follows:

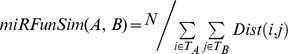
where *N* is the number of path in the protein interaction sub-network. The closer the miRNA targets are in the network, the higher the scores. The scores range from 0 to 1 and higher scores correspond to higher functional concordance between miRNAs.

### Performance Evaluation of miRFunSim

**Figure 2:**
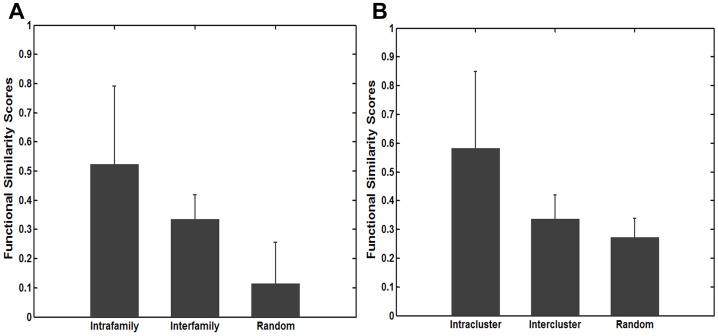
Performance evaluation of miRFunSim using miRNA family and miRNA cluster. (A) A comparison of functional similarity scores of intrafamily miRNA pairs, interfamily miRNA pairs and random miRNA pairs. (B) A comparison of functional similarity scores of intracluster miRNA pairs, intercluster miRNA pairs and random miRNA pairs.

The accumulating evidence revealed that miRNAs in the same family are likely to have similar functions [34], [35], [36]. Therefore, to evaluate the reliability of functional similarity scores computed by our miRFunSim method, we first downloaded miRNA family data from miRBase Database [30] and obtained 100 miRNAs whose target genes have been experimentally supported from TarBase [31]. Then we used our miRFunSim method to compute functional similarity scores of miRNA pairs between 100 miRNAs. These miRNA pairs were grouped into two classes: intrafamily miRNA pairs and interfamily miRNA pairs. We further compared the functional similarity scores of intrafamily miRNA pairs,interfamily miRNA pairs and random miRNAs pairs. As a result, the significant differences in functional similarity scores among intrafamily miRNA pairs, interfamily miRNA pairs and random miRNA pairs are observed (Figure 2A, Kruskal-Wallis test, df = 2, p-value = 0). The functional similarity scores for intrafamily miRNA pairs are significantly higher compared with interfamily miRNA pairs (p-value = 4.30e-5, Wilcoxon rank sum test) and random miRNA pairs (p-value = 1.80e-14, Wilcoxon rank sum test). Interfamily miRNA pairs also showed higher functional similarity scores than random miRNA pairs (p-value = 1.46e-20, Wilcoxon rank sum test).

It is well known that a large number of miRNAs have clustering propensity and tend to form some clusters. Previous studies have suggested that miRNA members within the same cluster are often located in a polycistron and display a homogeneous expression pattern [37], [38], which imply that these clustered miRNAs perhaps have common or similar functions. Therefore, we also computed functional similarity scores between miRNAs in the same cluster and between miRNAs not located in the same cluster using miRFunSim method (Fig. 2B). Statistical analyses showed that functional similarity scores among intracluster miRNA pairs, intercluster miRNA pairs and random miRNA pairs are also significantly different (Figure 2B, Kruskal-Wallis test, df = 2, p-value = 0). The functional similarity scores of intracluster miRNA pairs are significantly higher than those of intercluster miRNA pairs (p-value = 5.42e-6, Wilcoxon rank sum test) and random miRNA pairs (p-value = 1.40e-8, Wilcoxon rank sum test). Intercluster miRNA pairs also showed higher functional similarity scores than random miRNA pairs (p-value = 5.60e-23, Wilcoxon rank sum test). These results suggested that our method is reliable and sensible to measure the functional relationship between miRNAs. To investigate the robustness of our miRFunSim method, we first performed above analysis for the predicted miRNA target and examine whether our method is still able to measure the associations between miRNAs. The resulting scores between miRNAs using predicted targets are shown in Fig. S1A. The functional similarity scores for intrafamily and intracluster miRNA pairs are significantly higher compared with interfamily and intercluster miRNA pairs (p-value = 0 and p-value = 1.01e-6, Wilcoxon rank sum test). Then we further access our method by the removal of 5% and 10% network nodes in the protein interaction network randomly (Fig. S1B, C). As shown in Fig. S1B, C, the functional similarity scores of intrafamily and intracluster miRNA pairs are significantly higher than those of interfamily (5%:p-value = 9.47e-5 and 10%:p-value = 9.53e-5, Wilcoxon rank sum test) and intercluster miRNA pairs (p-value = 2.01e-3 and p-value = 1.6e-3, Wilcoxon rank sum test).

**Figure 3:**
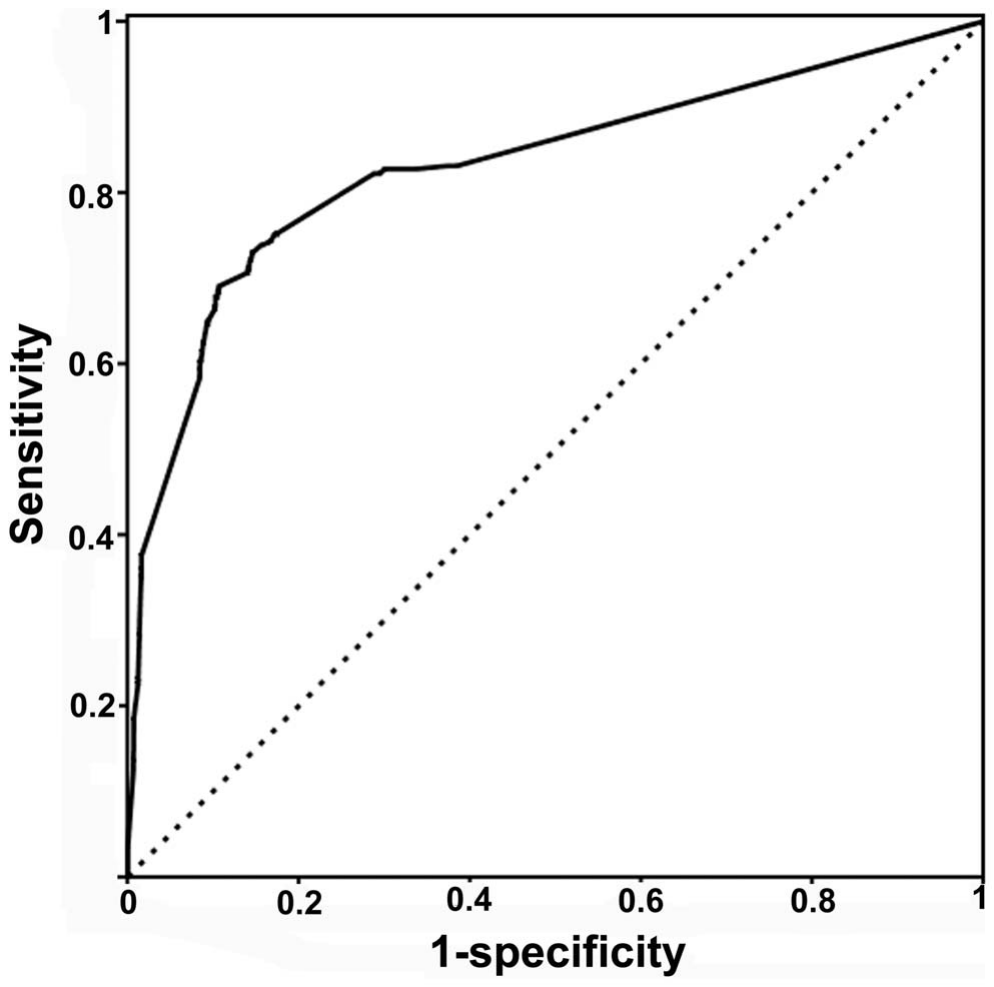
Area under ROC curve (AUC) analysis on 270 high-quality experimentally verified miRNA-disease associations from Jiang’s study and 100 miRNAs whose target genes have been experimentally supported using our miRFunSim method.

To further evaluate the performance of our miRFunSim method for quantifying the associations between two miRNAs, we performed a validation analysis on experimentally verified miRNA-disease associations. It has been proven that miRNAs with similar functions tend to be involved in phenotypically similar disease, and miRNAs associated with common diseases are more related in function [17], [33], [34]. Our validation analysis for performance of miRFunSim method was based on above notion. First, we obtained 270 high-quality experimentally verified miRNA-disease associations from Jiang’s study [33] and 100 miRNAs whose target genes have been experimentally supported. For each disease, the functional similarity score between every two miRNAs associated with this disease were computed using the miRFunSim method as the testing case. For each testing case, 99 simulated miRNA pairs were generated and the target genes of simulated miRNA pairs were randomly sampled from all human protein-coding genes keeping the same size as the given testing case. The functional similarity scores of 99 simulated miRNA pairs also were computed using the miRFunSim method as negative controls of the given testing case. Second, we prioritized the testing case together with 99 negative controls according to the scores derived from miRFunSim method. Therefore, for each testing case, we obtain a ranking list, that is, prioritization of 100 miRNA pairs. In total, we obtained 562 ranking lists, each with 100 prioritizations. Third, from 562 ranking lists, we calculated the sensitivity and specificity at varying thresholds. Sensitivity measures the proportion of the testing case whose ranking is higher than a given score. Specificity measures the proportion of negative controls ranked below this score. Finally, a receiver operating characteristics (ROC) curve was plotted by varying the score and the area under the curve (AUC) was calculated. We used AUC as a standard measure of the performance of miRFunSim. The maximum value of AUC is 100%, which indicates every testing case is ranked first in the ranking list. Figure 3 shows the results of performance evaluation of miRFunSim using the ROC curves obtained by calculating the sensitivity (sensitivity = TP/(TP +FN)) and 1-specificity (specificity = TN/(TN+FP)) by varying the threshold. Our miRFunSim method tested on 270 high-quality experimentally verified microRNA-disease associations achieved an AUC of 83.1%, suggesting that miRFunSim can recover the miRNA pairs associated with common disease and efficiently quantify the relationship between miRNAs.

### Comparisons with Other Existing Similar Methods

**Figure 4:**
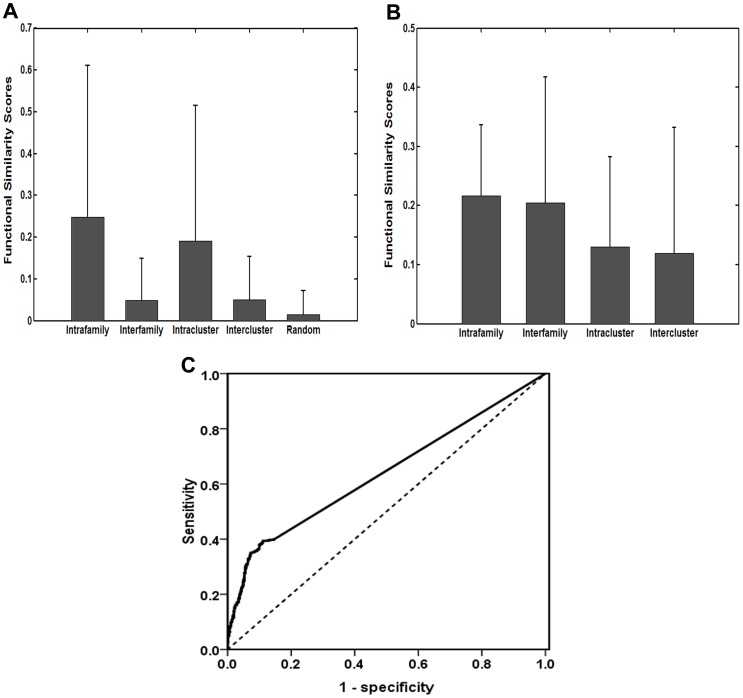
A performance comparison analysis between miRFunSim and other existing methods with similar functions. (A) The distribution and comparison of functional similarity scores of intrafamily, interfamily, intracluster,intercluster and random miRNA pairs computed by Yu’s method. (B) The distribution and comparison of functional similarity scores of intrafamily, interfamily, intracluster, intercluster miRNA pairs computed by MISIM method. (C) Area under ROC curve (AUC) analysis on 270 high-quality experimentally verified miRNA-disease associations from Jiang’s study and 100 miRNAs whose target genes have been experimentally supported using Yu’s method.

Recently, several approaches have been proposed for comparing miRNAs. Yu *et al.* developed a method to determine functional similarity of miRNAs by using their target genes GO semantic similarities [18]. However, this method perhaps sometimes produces disappointing results because of some GO limitations. Another existing method, called MISIM, is to measure the similarity of their associated disease directed acyclic graph (DAG) to compare two miRNAs. However, this method relies on miRNA-disease association data, and is difficult to achieve high reliability when little miRNA-disease association data is available [17]. Here, we also performed a performance comparison analysis between miRFunSim and these two similar methods using the same datasets. First, we used the method presented by Yu *et al.* and MISIM to compute functional similarity scores of miRNA pairs between 100 miRNAs whose target genes have been experimentally supported from TarBase [31]. Then these miRNA pairs also were grouped into four classes: intrafamily miRNA pairs, interfamily miRNA pairs, intracluster miRNA pairs and intercluster miRNA pairs. As shown in Fig. 4A, the functional similarity scores produced by Yu’s method are significantly different among intrafamily, interfamily and random miRNA pairs (Kruskal-Wallis test, df = 2, p-value = 0), and among intracluster, intercluster and random miRNA pairs (Kruskal-Wallis test, df = 2, p-value = 0). However, there is no significant difference in functional similarity scores produced by MISIM method between intrafamily and interfamily miRNA pairs (p-value = 0.25, Wilcoxon rank sum test), and between intracluster and intercluster miRNA pairs (p-value = 0.19, Wilcoxon rank sum test) (Fig. 4B), suggesting that the functional similarity scores produced by Yu’s method and our miRFunSim method can better reflect the functional relationship of miRNAs based on miRNA families and miRNA clusters than MISIM method. Next, we also tested Yu’s method on 270 high-quality experimentally verified miRNA-disease associations to compute the functional similarity score between every two miRNAs associated with the same disease, and obtained a ROC curve as the methods described in our analysis. Finally, the method presented by Yu *et al*. achieved an AUC of 63.9% (Fig. 4C), but is less than an AUC of 83.1% obtained by our miRFunSim method tested on the same datasets. Taken together, these results suggested that our miRFunSim method can achieve more effective and more reliable performance for quantifying the associations between miRNAs compared with other available similar methods.

### Case Study of Liver Cancer

As an example, to illustrate the application of quantifying the relationship between miRNAs using miRFunSim method, we presented a case study of liver cancer, which is one of the most common cancers, and applied the miRFunSim method to identify novel candidate liver cancer-related miRNAs. First, we retrieved 15 miRNAs which have been experimentally verified to contribute to the development of liver cancer and have experimentally verified target genes in TarBase as seed miRNAs. Next, we computed the functional similarity scores between every seed miRNAs and every miRNA from the remaining 85 miRNAs using miRFunSim method. The higher the score is, the more likely the miRNAs is associated with liver cancer. Finally, we prioritized all 1275 miRNA pairs for liver cancer according to their scores. The top 15 miRNA pairs with the highest functional similarity scores (score>0.5 and p<0.005) were chosen and 12 miRNAs with the highest functional similarity scores with seed miRNAs were listed as candidate liver cancer-related miRNAs and shown in Table 1. Among top 12 miRNAs, 8 miRNAs have been recorded to be deregulated in liver cancer and possibly contribute to the development of liver cancer, and 4 miRNAs (miR-15b,miR-20, miR-15 and miR-34) have been verified to be deregulated in other cancers in miR2Disease [39], and PhenomiR [37] databases which provide comprehensive resources for miRNA deregulation in disease. When our research is in progress, a new study provided further supporting evidence for one of the remaining four candidate liver cancer-related miRNAs. Li *et al.* [40] found that miR-34 participate in the neoplastic transformation of liver cancer stem cells (LCSCs) during hepatocarcinogenesis.

**Table 1:** The top 12 miRNAs with the highest functional similarity scores with known experimentally verified liver cancer-related miRNAs.

MiRNAname	Scores	P-value	Liver cancer-related miRNAs	References
miR-145	1.0	0.001	YES	[41]
miR-15b	1.0	<0.001	*	[42], [43]
miR-20	1.0	<0.001	*	[44]
miR-200a	1.0	<0.001	YES	[45], [46]
miR-200c	1.0	0.002	YES	[47]
miR-214	1.0	0.002	YES	[48], [49]
miR-222	1.0	<0.001	YES	[49], [50]
miR-17-5p	0.75	<0.001	YES	[51]
miR-125a	0.67	<0.001	YES	[45], [52]
miR-127	0.60	<0.001	YES	[53]
miR-15	0.55	<0.001	*	[54]
miR-34	0.51	<0.001	*	[55]

Note: The miRNAs which have been recorded to be deregulated in liver cancer in previous studies were designated as “YES”. The miRNAs which have been verified to be deregulated in other cancers in miR2Disease and PhenomiR databases were designated as “*”.

### Conclusions

In this study, we presented a novel computational framework and method, called miRFunSim, for quantifying the associations between miRNAs based on miRNAs targeting propensity and proteins connectivity in the integrated protein-protein interaction network. We applied the miRFunSim method to compare 100 miRNAs whose target genes have been experimentally supported from TarBase [31] and compared the distributions of functional similarity scores among intrafamily, interfamily and random miRNA pairs, and among intracluster, intercluster and random miRNA pairs. The functional similarity scores of miRNAs in the same family or in the same cluster are significantly higher compared with other miRNAs. These results suggested that the miRFunSim method can better reflect the functional similarities and differences of miRNA pairs in the different groups. We further tested miRFunSim method on 270 high-quality experimentally verified miRNA-disease associations to recover the known miRNA pairs associated with the same disease and achieved a higher AUC of 83.1%. In comparison with existing similar methods, our miRFunSim method can achieve more effective and more reliable performance for measuring the functional similarity of miRNAs. With the improvement in coverage of PPI network and in prediction accuracy of miRNA targets, the proposed miRFunSim method will perform better for quantifying the associations between miRNAs. Furthermore, this method can be extended to other species when PPIN data and targets of miRNAs are available.

## Supporting Information

Figure S1 **The robustness analysis results for measuring the relationship of miRNAs using miRFunSim.** (A) A comparison of functional similarity scores between intrafamily and interfamily miRNA pairs, and between intracluster and intercluster miRNA pairs using predicted miRNA targets. (B) A comparison of functional similarity scores between intrafamily and interfamily miRNA pairs, and between intracluster and intercluster miRNA pairs by the removal of 5% network nodes in the protein interaction network randomly. (C) A comparison of functional similarity scores between intrafamily and interfamily miRNA pairs, and between intracluster and intercluster miRNA pairs by the removal of 10% network nodes in the protein interaction network randomly.
(DOC)

File S1 **Information of experimentally verified miRNA targets from TarBase.**
(TXT)

File S2 **Information of predicted miRNA targets from starBase by at least three prediction algorithms with readNum> = 1 and biological complexity > = 1.**
(TXT)
