# Supplementary material for: Inferring Potential microRNA-microRNA Associations Based on Targeting Propensity and Connectivity in the Context of Protein Interaction Network
Source: PLoS One. 2013 Jul 16;8(7):e69719. doi: 10.1371/journal.pone.0069719 (PMC3713046; doi:10.1371/journal.pone.0069719)
Supplement: Figure S1 — The robustness analysis results for measuring the relationship of miRNAs using miRFunSim. (A) A comparison of functional similarity scores between intrafamily and interfamily miRNA pairs, and between intracluster and intercluster miRNA pairs using predicted miRNA targets. (B) A comparison of functional similarity scores between intrafamily and interfamily miRNA pairs, and between intracluster and intercluster miRNA pairs by the removal of 5% network nodes in the protein interaction network randomly. (C) A comparison of functional similarity scores between intrafamily and interfamily miRNA pairs, and between intracluster and intercluster miRNA pairs by the removal of 10% network nodes in the protein interaction network randomly. (DOC) [file pone.0069719.s001.doc]

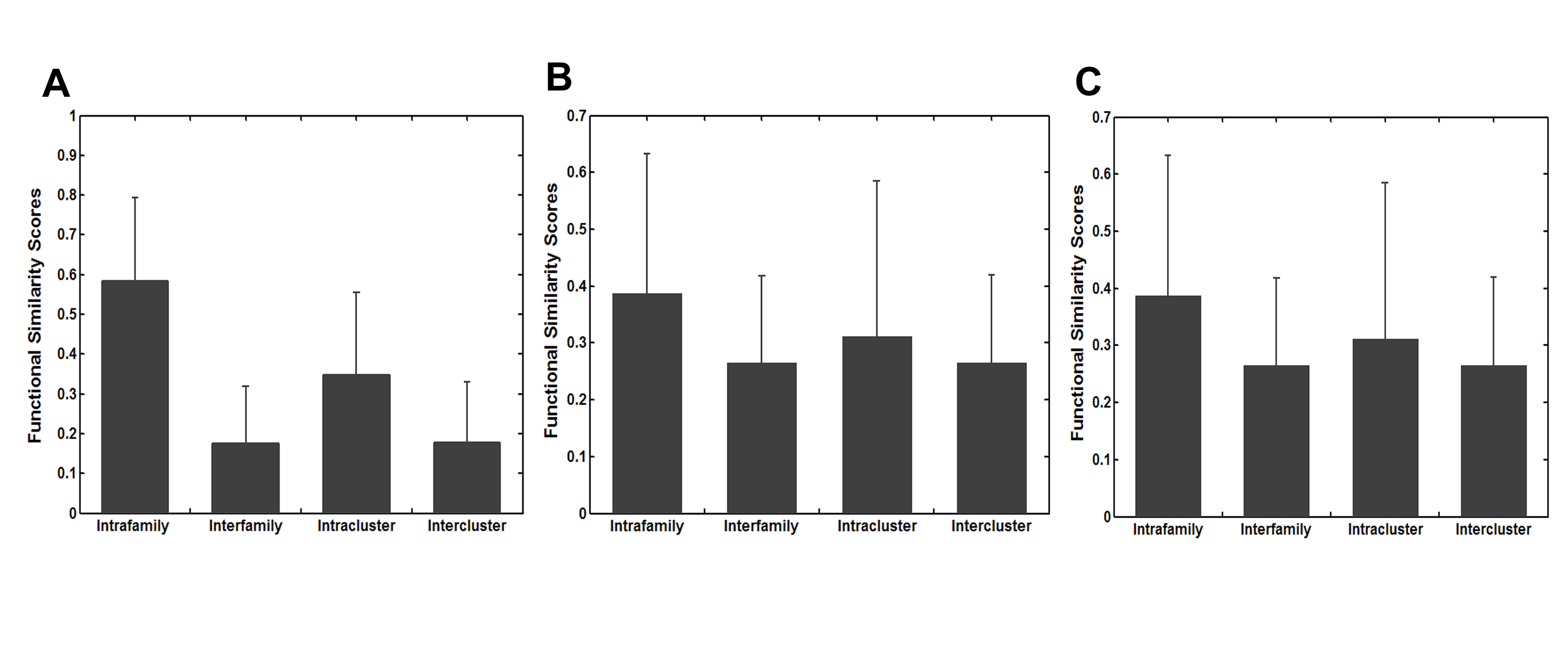


Figure S1. The robustness analysis results for measuring the relationship of miRNAs using miRFunSim. (A) A comparison of functional similarity scores between intrafamily and interfamily miRNA pairs, and between intracluster and intercluster miRNA pairs using predicted miRNA targets. (B) A comparison of functional similarity scores between intrafamily and interfamily miRNA pairs, and between intracluster and intercluster miRNA pairs by the removal of 5% network nodes in the protein interaction network randomly. (C) A comparison of functional similarity scores between intrafamily and interfamily miRNA pairs, and between intracluster and intercluster miRNA pairs by the removal of 10% network nodes in the protein interaction network randomly.
